# The Effects of Fine Particle Components on Respiratory Hospital Admissions in Children

**DOI:** 10.1289/ehp.11848

**Published:** 2008-12-16

**Authors:** Bart Ostro, Lindsey Roth, Brian Malig, Melanie Marty

**Affiliations:** Office of Environmental Health Hazard Assessment, California Environmental Protection Agency, Oakland, California, USA

**Keywords:** children, EC, hospital admissions, OC, PM_2.5_, respiratory, species

## Abstract

**Background:**

Epidemiologic studies have demonstrated an association between acute exposure to ambient fine particles and both mortality and morbidity. Less is known about the relative impacts of the specific chemical constituents of particulate matter < 2.5 μm in aerodynamic diameter (PM_2.5_) on hospital admissions.

**Objective:**

This study was designed to estimate the risks of exposure to PM_2.5_ and several species on hospital admissions for respiratory diseases among children.

**Data and Methods:**

We obtained data on daily counts of hospitalizations for children < 19 and < 5 years of age for total respiratory diseases and several subcategories including pneumonia, acute bronchitis, and asthma for six California counties from 2000 through 2003, as well as ambient concentrations of PM_2.5_ and its constituents, including elemental carbon (EC), organic carbon (OC), and nitrates (NO_3_). We used Poisson regression to estimate risks while controlling for important covariates.

**Results:**

We observed associations between several components of PM_2.5_ and hospitalization for all of the respiratory outcomes examined. For example, for total respiratory admissions for children < 19 years of age, the interquartile range for a 3-day lag of PM_2.5_, EC, OC, NO_3_, and sulfates was associated with an excess risk of 4.1% [95% confidence interval (CI), 1.8–6.4], 5.4% (95% CI, 0.8–10.3), 3.4% (95% CI, 1.1–5.7), 3.3% (95% CI, 1.1–5.5), and 3.0% (95% CI, 0.4–5.7), respectively. We also observed associations for several metals. Additional associations with several of the species, including potassium, were observed in the cool season.

**Conclusion:**

Components of PM_2.5_ were associated with hospitalization for several childhood respiratory diseases including pneumonia, bronchitis, and asthma. Because exposure to components (e.g., EC, OC, NO_3_, and K) and their related sources, including diesel and gasoline exhaust, wood smoke, and other combustion sources, are ubiquitous in the urban environment, it likely represents an identifiable and preventable risk factor for hospitalization for children.

Fine particles (particles < 2.5 μm in aerodynamic diameter; PM_2.5_) have been linked to a variety of poor health outcomes, including mortality and cardiorespiratory morbidity (Dominici et al. 2006; Ostro et al. 2006; Schwartz et al. 1996). However, PM_2.5_ encompasses a chemically heterogeneous mix of solid and liquid particles emitted from a variety of sources, and it is likely that the different chemical species within PM_2.5_ will have varying effects on specific health end points. Several epidemiologic studies have attempted to address this question, looking at the effects of specific fine particle species on mortality (Burnett et al. 2000; Franklin et al. 2008; Laden et al. 2000; Ostro et al. 2007). However, studies such as those by Burnett et al. (1995) and Peel et al. (2005) examining associations between specific PM_2.5_ constituents and morbidity outcomes such as hospital admissions, particularly among children, have been less common. With regulatory agencies actively targeting PM constituents for pollution control, a greater understanding of the impacts of current and future control efforts is needed.

Children are a demographic of special concern, as they represent a population at increased risk for air pollution–related respiratory conditions because of their biologic characteristics (e.g., immature lungs and immune system, higher breathing rates) and behavior (e.g., more time spent outdoors) (Miller et al. 2002; Schwartz 2004). Particle deposition, specifically, is higher in both young children and asthmatics (Chalupa et al. 2000; Ginsberg et al. 2005), and several studies have observed associations between PM_2.5_ and bronchitis and asthma exacerbation in children (Barnett et al. 2005; McConnell et al. 2003). Thus, when designing and instituting pollution controls, the impacts on children from exposure to components of PM_2.5_ need to be documented.

Our study used speciated PM_2.5_ monitoring data to examine the association between PM_2.5_ and its constituents with respiratory hospitalizations among children 0–18 years of age in six California counties. Our goal is to provide further insight into the relative toxicity of fine particle constituents, which would generate multiple benefits. For example, such an analysis would identify specific agents of greater concern within particulate matter (PM), which could be more thoroughly investigated on a mechanistic level in toxicology studies. Second, it would help identify which communities may be at greater risk because of their specific constituent mix of PM_2.5_. Finally, it could implicate certain fine particle sources as posing a greater health threat based on the chemical profile of the fine particles they emit. In fact, certain constituents can be used as indicators of PM_2.5_ specific to sources such as gasoline and diesel exhaust, crustal material, and fuel combustion from stationary sources such as power plants, wood smoke, and other biomass burning.

## Data and Methods

### Hospitalization data

We obtained data for all hospitalizations in California from the Office of Statewide Health Planning and Development, Healthcare Quality and Analysis Division (Sacramento, CA), for the period from 1 January 2000 through 31 December 2003. Based on the *International Classification of Diseases, 9th Revision* (ICD-9) (World Health Organization 1975), hospital admissions for children < 19 years of age were classified into one or more categories: all respiratory disease (ICD-9 codes 460–519), asthma (ICD-9 code 493), acute bronchitis (ICD-9 code 466), and pneumonia (ICD-9 codes 480–486). Hospital admission counts were then aggregated based on the child’s reported county of residence to yield a daily time-series of relevant hospitalizations for each county. For total respiratory admissions, we further stratified by ages < 5 years and 5–18 years of age. Finally, we examined the same outcomes for the cool season, defined here as October through March.

### Pollutant and meteorologic data

Available PM_2.5_ speciation data were obtained for 2000–2003 from the California Air Resources Board (Sacramento, CA). The speciation monitors were filter-based Met One Speciation Air Sampling Systems (SASS) (Met One Instruments Inc., Grants Pass, OR) belonging to the Speciation Trends Network (STN). Counties were included if there were ≥ 180 days of observations with PM_2.5_ species data during the study period, which limited the study to six California counties with a sufficient duration of speciated PM_2.5_ monitoring. These counties encompass approximately 8.7 million people, or roughly 25% of the state’s population. In Kern, Riverside, and Santa Clara Counties, speciation data from two co-located monitors were available. Fresno, Sacramento, and San Diego Counties each had one monitor with relevant data. Speciation data were generally recorded every 3 days. The number of available days of data over the 4-year period ranged from 227 (San Diego) to 381 (Sacramento), because of varying monitor start dates. The components of PM_2.5_ were measured as 24-hr averages and included elemental carbon (EC), organic carbon (OC), nitrates (NO_3_), sulfates (SO_4_), copper (Cu), iron (Fe), potassium (K), silicon (Si), and zinc (Zn). These species were chosen because they *a*) include the larger components of PM_2.5_ in our study sample; *b*) had a signal-to-noise ratio of ≥ 2, and *c*) had a majority of their values greater than the detection limit for each county monitor used. Signal-to-noise ratio for a species was defined as


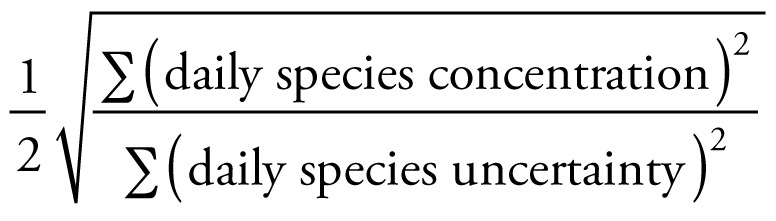


(Rubin et al. 2006).

To adjust for the potential confounding between temperature and hospital admissions, average daily temperature and relative humidity data were collected at meteorologic stations at each of the counties using data provided by the California Air Resources Board (unpublished data) or the California Irrigation Management Information System (wwwcimis.water.ca.gov/cimis/data.jsp). The daily hospital admission counts and pollution and meteorologic data were integrated using SAS (version 9.1; SAS Institute Inc., Cary, NC).

### Methods

The daily hospital admission counts are non-negative discrete integers that correspond to rare events. Therefore, our analysis used Poisson regression conditional on the explanatory variables: time, day of the week, temperature, relative humidity, and pollutant. Similar model specifications were used for each county and included natural spline smoothers for both a daily time trend and meteorology. The natural spline fits multiple parametric cubic functions evenly placed over the range of the variable, such that controlling the total number of fitting functions, or degrees of freedom (df), controls the smoothness of the resulting spline. The smoothing of the relationship between time and hospital admissions helps to control for the effects of seasonality and other factors that are time dependent. Issues and sensitivity of model and knot selection have been discussed previously (Ostro et al. 2006).

The explanatory variables were day of the week, smoothed splines of daily unlagged average temperature and humidity (each with 3 df), and a smoothing spline of time with 4 df per calendar year. The lags and degrees of freedom for meteorologic variables were chosen based on their effectiveness in controlling for weather variables and promoting best model fits in both this analysis and previous studies (Basu et al. 2008; Ostro et al. 2006). Similarly, 4 df were chosen for the time spline *a priori* because this number controls well for seasonal and secular patterns (Ostro et al. 2006). For each county, each species was then examined separately in a model using single-day lags of 0–3. Analyses looking at cool season (October–March) effects followed the same structure but included a binary indicator for season (warm vs. cool) and an interaction variable for season and pollutant. County-specific results were later combined in a meta-analysis, using a random-effects model to help account for heterogeneity between county estimates (DerSimonian and Laird 1986).

In contrast to time-series studies with consecutive daily data, different lags in our analyses will relate to different days in the time series of hospital admissions. For example, an unlagged analysis compares pollutant readings with admission counts occurring on the pollutant sampling days. An analysis using 1-day lags would compare those same pollutant readings to admissions occurring 1 day after the sampling day. So although our pollutant data are not available on a daily basis, the daily outcome data afford us the ability to examine different single-day lags.

We calculated the results using R (version 2.6.2; R Development Core Team 2006) for the single-county and meta-analyses. To compare relative impacts of the observed concentrations of the species components of PM_2.5_, we present the results as the percent excess risk (ER) in daily hospital admissions for the interquartile range (IQR = 75th–25th percentile) of daily exposure for each species, where ER per IQR = [(e*^beta^* ^× IQR^) – 1] × 100 and *beta* is the combined regression coefficient from the meta-analysis.

## Results

Table 1 provides descriptive statistics about the county characteristics, hospitalizations, PM_2.5_ and its components, and meteorologic data. As displayed in Table 1, the average number of daily respiratory hospital admissions for children < 19 years of age ranged from 3.9 in Kern County to 10.2 in Riverside County, with children < 5 years of age generating about 75% of the admissions. Mean daily PM_2.5_ concentrations during the study period averaged 19 μg/m^3^ with a range of 15 μg/m^3^ in Sacramento to 29 μg/m^3^ in Riverside County, which exceed the U.S. Environmental Protection Agency annual average PM_2.5_ standard of 15 μg/m^3^ in five of the six counties, and the California annual average standard of 12 μg/m^3^ in all six counties.

Table 2 shows the number of useable observations, mean, and IQR of each component of PM_2.5_. In this data set, PM_2.5_ is dominated by EC, OC, NO_3_, and SO_4_ which are 5%, 36%, 28%, and 10% of the total, respectively. We generally found moderate correlations (*r* < 0.5) among the species, whereas we observed high correlations between PM_2.5_ and EC, OC, and NO_3_ (0.7, 0.8, and 0.9, respectively).

Table 3 summarizes the empirical relationships between the health end points and the various lags of PM_2.5_ and its species. Many species were associated with total hospitalizations for respiratory disease, particularly for those < 5 years of age. We observed associations with multiple lags of PM_2.5_, OC, NO_3_, and Fe, and significant associations for EC, SO_4_, Cu, and Si. Single-day lags of 1–3 days were usually more important than unlagged values. The disease-specific results suggest that PM_2.5_ and its components were most associated with acute bronchitits and pneumonia, but we observed associations for asthma admissions as well. Analyses of the cool season indicated similar associations between the species and the various health end points. In addition, additional associations were observed between K and admissions for all respiratory disease as well as with pneumonia and bronchitis.

**Figure 1:**
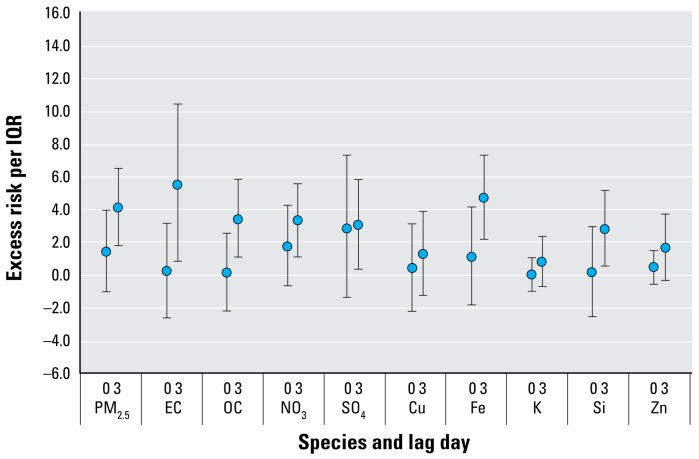
Association of PM_2.5_ species with total respiratory admissions for those < 19 years of age (percent excess risk per IQR and 95% CIs).

Figure 1 summarizes the excess risks for all-year respiratory hospital admissions among children < 19 years of age. For ease in interpretation, only single-day lags of 0 and 3 are presented in the figures. With collection of species data every third day, these lags correspond to the same count day of hospital admissions. However, additional details on other lags are provided in the Supplemental Material (online at http://www.ehponline.org/members/2008/11848/suppl.pdf). For a 3-day lag, excess risks of 3–6% for the IQR were observed for many of the PM_2.5_ species, with the highest risks observed for PM_2.5_, EC, and Fe with respective excess risks of 4.1% [95% confidence interval (CI), 1.8–6.4], 5.4% (95% CI, 0.8–10.3), and 4.7% (95% CI, 2.2–7.2), respectively. Excess risks of approximately 3% were observed for OC, NO_3_, SO_4_, and Si.

**Figure 2:**
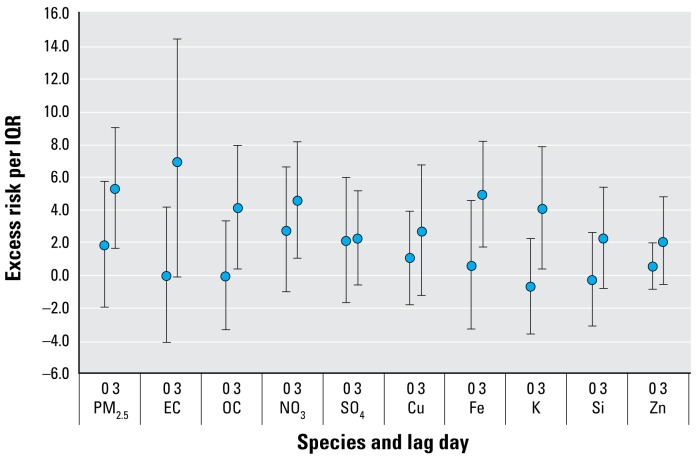
Association of PM_2.5_ species with total respiratory admissions during the cool season for those < 19 years of age (percent excess risk per IQR and 95% CIs).

Figure 2 summarizes the excess risks for respiratory hospital admissions during the cool season for those < 19 years of age. Again, higher effect estimates per species IQR were observed for PM_2.5_, EC, and Fe compared with other species, with the excess risks using the same 3-day lag in the cool season being 5.1% (95% CI, 1.6 to 8.9), 6.8% (95% CI, –0.2 to 14.2), and 4.8% (95% CI, 1.7 to 8.0), respectively. Except for SO_4_, other species associated with juvenile respiratory admissions in full-year analyses remained significant in cool season analyses, with excess risks per IQR generally falling around 4%. Similarly, K, a species not associated with admissions in full-year analyses, had a significant excess risk of 4.0% (95% CI, 0.3–7.7) during the cool season.

## Discussion

Our analysis of hospital admissions for children indicates that PM_2.5_ and several of its components have important effects on hospital admissions for respiratory diseases, especially acute bronchitis and pneumonia. Acute exposure to total PM_2.5_ was associated with total respiratory hospital admissions for those < 19 years and < 5 years of age. Children < 5 years of age were particularly sensitive to PM_2.5_ and several of its components including EC, OC, NO_3_, SO_4_, Fe, and Si. We observed excess risks of 3–7% for many of the respiratory outcomes, based on species IQR. We observed similar risks during the cool season, where associations with K were also demonstrated.

The associations demonstrated for total PM_2.5_ levels corroborate previous studies that observed effects of fine PM on respiratory hospitalizations in children (Barnett et al. 2005; Lin et al. 2002; van der Zee et al. 1999). Earlier studies also found associations between PM < 10 μm in aerodynamic diameter (PM_10_) and total respiratory, asthma, and pneumonia hospitalizations (Pope 1989; Tolbert et al. 2000). Despite the direct causes of pneumonia and other respiratory infections being biologic in nature, the effect of air pollution on the development of severe cases requiring hospitalization is especially plausible in children, because particulates likely hamper the ability of an already immature immune system to clear bacteria and other pathogens from the lung (Dietert et al. 2000). Other studies have also indicated that short-term incidences of respiratory disease may not be the only impact on children’s health. Lung development itself may be compromised, as PM_2.5_ and, more specifically, EC were among pollutants strongly associated with decreased lung function attainment in school children (Gauderman et al. 2004).

These studies of children also complement previous studies on elderly populations reporting associations between PM_2.5_ and PM_10_ levels and hospitalizations for respiratory diseases, including respiratory tract infections, chronic obstructive pulmonary disease, and pneumonia (Dominici et al. 2006; Zanobetti et al. 2000).

Because data collection on the chemical components of PM_2.5_ has only recently begun, however, fewer studies are available on the effects related to specific species. Most of these studies link specific components of PM_2.5_ to mortality. For example, in analyses of six California counties and of Phoenix, Arizona, acute exposure to several of these components, including EC, OC, Fe, Zn, and K, was associated with increases in daily counts of mortality (Mar et al. 2004; Ostro et al. 2007). In contrast, previous epidemiologic studies looking at fine particle constituents and morbidity have been sparse. Associations between OC and emergency department visits for pneumonia have been reported (Peel et al. 2005), as well as asthma attacks in children and exhaled nitric oxide, a biomarker for airway inflammation (Delfino et al. 2003, 2006; Peel et al. 2005; Sinclair and Tolsma 2004). A recent study of pre-school-age children in the Czech Republic reported associations between bronchitis and both PM_2.5_ and polycyclic aromatic hydrocarbons (PAHs) (Hertz-Picciotto et al. 2007).

To date, studies focusing on NO_3_ and SO_4_ have been mixed in their findings (Schlesinger et al. 2006). We observed associations between daily exposure to NO_3_ and respiratory disease for both those < 19 years and those < 5 years of age and more modest associations between SO_4_ and acute bronchitis. NO_3_ constitute a significantly larger, and SO_4_ a smaller, share of PM_2.5_ in California than observed in most of the rest of the United States (Bell et al. 2007). However, because of lack of available data, NO_3_ have not been extensively examined in epidemiologic studies of air pollution. In one of the few previous studies examining the association between acute exposure to SO_4_ and respiratory morbidity in children, an association was observed for children with chronic respiratory symptoms between SO_4_ and both lower respiratory tract symptoms and decrements in lung function (van der Zee et al. 1999). In addition, Burnett et al. (1995) reported associations between SO_4_ and respiratory hospital admissions for children < 15 years of age among hospitals in the province of Ontario, Canada, and Sarnat et al. (2008) found associations between emergency department visits for respiratory disease with SO_4_, but not with any other species, in an Atlanta-based study. Although motor vehicle fuel combustion sources of NO_3_ are, of course, ubiquitous throughout the state, there are only a few major sources of SO_4_ in California, because most state utilities do not use either coal or oil. These sources are found primarily in the Los Angeles Air Basin and are associated with the local port facilities. Major SO_4_ sources include petroleum refining (both fuel combustion and industrial processes), heavy duty diesel trucks, and fuel combustion of ships and commercial boats (California Air Resources Board 2007).

We also found associations between hospital admissions and several metals including Cu, Fe, and, to a lesser extent, Zn. Existing studies indicate that Fe is a marker for brake wear and is found in road dust and soil, and Cu and Zn may be generated from vehicular-related sources including brake wear, emissions from lubrication oil, and tire wear (Schauer et al. 2006). Several human and animal studies have reported associations of ferric iron and other metallic compounds with the generation of oxidative stress and pulmonary inflammation (Ghio 2004; Pritchard et al. 1996), and another study in laboratory rats demonstrated that pulmonary Fe exposure impaired both immune function and the ability to clear bacteria (Zelikoff et al. 2002). Burnett et al. (2000) reported associations between mortality and daily exposures to several metals, including Zn, nickel, and Fe in their study of eight Canadian cities. In addition, Hirshon et al. (2008) found associations between ambient Zn and asthma urgent care visits and hospitalization among a cohort of individuals < 18 years of age. Intratracheal instillation studies in rodents demonstrated that pulmonary neutrophilic inflammation and protein leakage could be partially reproduced by zinc sulfate and copper sulfate, both major transition metals in the PM samples (Gottipolu et al. 2008; Kodavanti et al. 2002). Cu itself induced pulmonary inflammation, and in combination with Zn produced higher numbers of total lavageable cells and neutrophils than either treatment alone (Gottipolu et al. 2008). Our findings for Si are supported by evidence that crystalline silica is a well-known occupational fibrosis hazard and can produce oxidative stress, lung inflammation, and cell death at relatively low exposures (Kaewamatawong et al. 2006).

Although the above evidence presents the case that specific constituents cause direct effects, our study examines levels of PM_2.5_ constituents under real-world conditions, and our observed effects for one constituent may be attributable to its correlation with other constituents from a similar generating source. Source apportionment was not performed in this current analysis; however, a study of source apportionment methods found that correctly selected single-species tracers can be almost as informative in identifying source contributions as more rigorous methods (Sarnat et al. 2008).

EC, the tracer for diesel particulates used by Sarnat et al. (2008), is likely a reasonable marker for diesel exhaust in this study as well. Because there are few other sources of primary emissions of carbonaceous material such as coal and fuel oil combustion in California, approximately 80% or more of the EC is derived from diesel exhaust (Schauer and Cass 2000). Existing toxicologic and human clinical studies (Salvi et al. 1999) indicate that diesel exhaust particles induce both pulmonary inflammation and cell damage, including death of alveolar macrophages, through the generation of reactive oxygen species (Hiura et al. 2000; Mar et al. 2000; Nel et al. 2001). Alveolar macrophage death may lessen defense against pulmonary pathogens. Atopic individuals challenged intranasally with diesel exhaust particles and an allergen exhibited increased antigen-specific immunoglobulin E, cytokine, and chemokine levels and increased mast cell degranulation, indicating enhanced allergic inflammation, the principal hallmark of asthma (Diaz-Sanchez et al. 1997, 2000; Nel et al. 1998). So in relation to the EC associations observed in our study, EC could be serving as a surrogate for the larger category of diesel exhaust particles, which, in addition to EC, can include trace metals or adherent organic hydrocarbons such as PAHs, any or all of which could be acting to cause physiologic effects (Li et al. 2003).

Other studies apportioning ambient PM_2.5_ levels to sources suggest that acute exposure to particles from motor vehicle exhaust, fossil fuel combustion, and vegetative burning—all sources marked by emissions of EC and OC (Sarnat et al. 2008)—is associated with both respiratory morbidity (Andersen et al. 2007) and daily mortality (Laden et al. 2000; Mar et al. 2000).

Our findings suggesting effects for metals, NO_3_, SO_4_, EC, and OC implicate man-made combustion sources (i.e., emissions from motor vehicle and industrial fuel combustion, refineries, and industrial processes) and related processes (i.e., brake and tire wear) as significant producers of bioactive fine particulate. K, generally regarded as a marker for wood and biomass burning (Khalil and Rasmussen 2003; Laden et al. 2000; Sarnat et al. 2008), was not consistently related to respiratory admissions in our full-year analyses. However, cold season–specific K levels were significantly associated with respiratory admissions, implying that fine particles from wood smoke may also contribute to respiratory morbidity, as has been suggested in previous research (Naeher et al. 2007). Si is generally regarded as a good tracer for crustal or soil particles (Laden et al. 2000; Marcazzan et al. 2001), and the significant associations we found between Si and respiratory admissions suggest that crustal particulates can also affect respiratory health.

We found that for many of the respiratory outcomes, stronger associations tended to occur with a 1- to 3-day lag of the species. Although there is little to justify an appropriate lag structure *a priori* for the vast majority of PM_2.5_ constituents, it is of note that Peel et al. (2005) also found that lags of several days were significant for respiratory ER visits for children. Additional support for longer lags for respiratory disease outcomes is provided by two multicity studies, where hospital admissions for pneumonia were more strongly associated with lags of 2 days or more (Braga et al. 2001; Zeka et al. 2005). In our analysis, as in the others cited, it is unclear whether the longer lag associations are attributable to biologic mechanisms or simply to stochastic variability. However, it is plausible that a latency of several days may occur between exposure and exacerbation of infection because of inflammation and immune suppression. In addition, there may be a delay between exposure and actual day of hospital admission, as opposed to an emergency department visit.

It is important to note the limitations of our study. First, spatial coverage of the monitors is limited for several of the counties. This is likely to introduce random measurement error into the analysis and the potential for downwardly biasing the effect estimates. In addition, differences in measurement techniques or spatial heterogeneity among constituents could introduce different levels of measurement error. Second, this study shares a shortcoming with other particulate studies relying on nondaily data. Because readings are taken every 3 days, comparisons of effect between lags are less direct than they would be with daily data. For this analysis, a same-day lag analysis compares measurements with hospitalizations that occurred on days when readings were taken. A 1-day lag analysis looks at the association between those same readings and all hospitalizations taking place 1 day after a measurement day, or what is in essence a different risk set. This introduces a greater possibility of chance differences in comparisons between different lags. Third, given the numbers of pollutants and end points examined and the relatively low number of observations, it is possible that some of the results may have occurred by chance or that the power to detect effects is limited. This likelihood is diminished somewhat by using a multicounty study design, which minimizes the likelihood that an unusual chance finding observed in one geographic area would heavily influence our conclusions. Also, in view of previous studies, our results seem quite plausible in terms of both the end points and the components for which associations were observed. Finally, associations for a given component may reflect its own toxicity or the toxicity of a correlated, measured or unmeasured, co-pollutant. Therefore, some caution is justified in attributing effects to any single component.

Our findings indicate that hospital admissions for childhood respiratory disease, especially pneumonia, are associated with exposure to PM_2.5_ and several of its components. Because these components and their related sources, including diesel exhaust, motor vehicle emissions, biomass burning, and other fuel combustion processes, are ubiquitous in the urban environment, small reductions in their concentrations could have a noticeable effect on childhood respiratory morbidity.

## Figures and Tables

**Table 1:** Mean county daily hospital admissions per day by morbidity category and air quality and meteorologic data, 2000–2003.

	Fresno	Kern	Riverside	Sacramento	San Diego	Santa Clara
Hospitalizations admission category (age)						
Respiratory (< 19 years)	6.1	3.9	10.2	4.8	9.8	6.1
Respiratory (< 5 years)	4.7	3.0	7.7	3.5	7.1	4.8
Respiratory (5–18 years)	1.4	0.9	2.5	1.3	2.7	1.3
Asthma (< 19 years)	1.7	0.8	2.4	1.3	2.8	1.4
Pneumonia (< 19 years)	1.5	1.7	3.6	1.4	2.7	1.7
Acute bronchitis (< 19 years)	1.9	0.8	2.6	1.2	2.2	1.7
County characteristics						
Population (1,000s)	799	662	1,545	1,223	2,814	1,683
Mean temperature (°F)	64.7	65.5	65.4	61.0	61.4	57.5
Mean relative humidity (%)	56.6	57.7	58.7	68.7	74.2	68.0
Mean PM_2.5_	22.4	21.4	28.8	14.8	15.6	15.0
No. of days in analysis	373	283	279	381	227	324

**Table 2:** Descriptive statistics for air pollutant species in California counties, 2000–2003.

	No.^a^	Full-year mean (μg/m^3^ )	Full-year IQR (μg/m^3^ )	Cool season mean (μg/m^3^ )	Cool season IQR (μg/m^3^ )
PM_2.5_	1,867	19.4	14.6	24.8	21.5
EC	1,867	1.0	0.8	1.4	1.2
OC	1,867	7.1	4.5	9.3	6.3
NO_3_	1,805	5.4	5.7	7.3	8.1
SO_4_	1,805	2.0	1.5	1.5	1.2
Cu	1,869	0.0077	0.0074	0.0080	0.0081
Fe	1,869	0.13	0.10	0.14	0.12
K	1,869	0.12	0.08	0.14	0.11
Si	1,869	0.18	0.15	0.15	0.13
Zn	1,869	0.012	0.010	0.018	0.014

a Total number of days across the six counties.

**Table 3:** Single-day lags asssociated with respiratory admissions by cause and age.

	Respiratory (< 19 years)	Respiratory (< 5 years)	Respiratory (5–18 years)	Asthma (< 19 years)	Pneumonia (< 19 years)	Acute bronchitis (< 19 years)
Full year						
PM_2.5_	1**, 3**	1*, 2*, 3**	1**, 3*		1*	1**, 3**
EC	3**	3**		3*		
OC	1**, 3**	1*, 3**			1**, 3*	1**, 3**
NO_3_	1**, 3**	3**	1*, 3*	1*		3*
SO_4_	3**	1*, 3**				3**
Cu	2**	2**	2*		2**	
Fe	1**, 3**	1*, 3**		0*, 1**, 3*	3**	
K			2*			
Si	3**	3**		1*	3*	2**
Zn				3*	0**	
Winter						
PM_2.5_	1**, 3**	1**, 2*, 3**			1*	1**, 3*
EC	3*	3*				3*
OC	1*, 3**	3**			1**	1**, 3**
NO_3_	1**, 3**	3**	1*	1**, 3**		
SO_4_	1*				0*	
Cu	1**, 2**	1**, 2**		1**	2**	1*
Fe	1*, 3**	1*, 3**		1*		
K	1**, 2*, 3**	1**, 2*, 3**			1**, 3*	1**
Si	2*	1*, 2**		1*		2**
Zn					0**	

* Single-day lag significant at *p* < 0.10.

** Single-day lag significant at *p* < 0.05.

## References

[b1-ehp-117-475] Andersen ZJ, Wahlin P, Raaschou-Nielsen O, Scheike T, Loft S (2007). Ambient particle source apportionment and daily hospital admissions among children and elderly in Copenhagen. J Expo Sci Environ Epidemiol.

[b2-ehp-117-475] Barnett AG, Williams GM, Schwartz J, Neller AH, Best TL, Petroeschevsky AL (2005). Air pollution and child respiratory health: a case-crossover study in Australia and New Zealand. Am J Respir Crit Care Med.

[b3-ehp-117-475] Basu R, Feng WY, Ostro BD (2008). Characterizing temperature and mortality in nine California counties. Epidemiology.

[b4-ehp-117-475] Bell ML, Dominici F, Ebisu K, Zeger SL, Samet J (2007). Spatial and temporal variation in PM_2.5_ chemical composition in the United States for health effects studies. Environ Health Perspect.

[b5-ehp-117-475] Braga A, Zanobetti A, Schwartz J (2001). The lag structure between particulate air pollution and respiratory and cardiovascular deaths in 10 US cities. J Occup Environ Med.

[b6-ehp-117-475] Burnett RT, Brook J, Dann T, Delocla C, Philips O, Cakmak S (2000). Association between particulate- and gas-phase components of urban air pollution and daily mortality in eight Canadian cities. Inhal Toxicol.

[b7-ehp-117-475] Burnett RT, Dales R, Krewski D, Vincent R, Dann T, Brook J (1995). Associations between ambient particulate sulfate and admissions to Ontario hospitals for cardiac and respiratory diseases. Am J Epidemiol.

[b8-ehp-117-475] California Air Resources Board (2007). 2007 Almanac Data, Oxides of Sulfur Projected Emission Inventory.

[b9-ehp-117-475] Chalupa D, Morrow P, Oberdorster G, Utell M, Frampton M (2000). Ultrafine particle deposition in subjects with asthma. Environ Health Perspect.

[b10-ehp-117-475] Delfino RJ, Gone H, Linn WS, Pellizzari ED, Hu Y (2003). Asthma symptoms in Hispanic children and daily ambient exposures to toxic and criteria air pollutants. Environ Health Perspect.

[b11-ehp-117-475] Delfino RJ, Staimer N, Gillen D, Tjoa T, Sioutas C, Fung K (2006). Personal and ambient air pollution is associated with increased exhaled nitric oxide in children with asthma. Environ Health Perspect.

[b12-ehp-117-475] DerSimonian R, Laird N (1986). Meta-analysis in clinical trials. Control Clin Trials.

[b13-ehp-117-475] Diaz-Sanchez D, Penichet-Garcia M, Saxon A (2000). Diesel exhaust particles directly induce activated mast cells to degranulate and increase histamine levels and symptom severity. J Allergy Clin Immunol.

[b14-ehp-117-475] Diaz-Sanchez D, Tsien A, Fleming J, Saxon A (1997). Combined diesel exhaust particulate and ragweed allergen challenge markedly enhances human in vivo nasal ragweed-specific IgE and skews cytokine production to a T helper cell 2-type pattern. J Immunol.

[b15-ehp-117-475] Dietert RR, Etzel RA, Chen D, Halonen M, Holladay SD, Jarabek AM (2000). Workshop to identify critical windows of exposure for children’s health: immune and respiratory systems work group summary. Environ Health Perspect.

[b16-ehp-117-475] Dominici F, Peng RD, Bell ML, Pham L, McDermott A, Zeger SL (2006). Fine particulate air pollution and hospital admission for cardiovascular and respiratory diseases. JAMA.

[b17-ehp-117-475] Franklin M, Koutrakis P, Schwartz P (2008). The role of particle composition on the association between PM_2.5_ and mortality. Epidemiology.

[b18-ehp-117-475] Gauderman WJ, Avol E, Gilliland F, Vora H, Thomas D, Berhane K (2004). The effect of air pollution on lung development from 10 to 18 years of age. N Engl J Med.

[b19-ehp-117-475] Ghio AJ (2004). Biological effects of Utah Valley ambient air particles in humans: a review. J Aerosol Med.

[b20-ehp-117-475] Ginsberg G, Foos B, Firestone M (2005). Review and analysis of inhalation dosimetry methods for application to children’s risk assessment. J Toxicol Environ Health A.

[b21-ehp-117-475] Gottipolu R, Landa E, Schladweiler M, Mcgee J, Ledbetter A, Richards J (2008). Cardiopulmonary responses of intratracheally instilled tire particles and constituent metal components. Inhal Toxicol.

[b22-ehp-117-475] Hertz-Picciotto I, Baker RJ, Yap PS, Dostal M, Joad JP, Lipsett M (2007). Early childhood lower respiratory illness and air pollution. Environ Health Perspect.

[b23-ehp-117-475] Hirshon J, Shardell M, Alles S, Powell J, Squibb K, Ondov J (2008). Elevated ambient air zinc increases pediatric ashma morbidity. Environ Health Perspect.

[b24-ehp-117-475] Hiura T, Li N, Kaplan R, Horwitz M, Seagrave J, Nel A (2000). The role of a mitochondrial pathway in the induction of apoptosis by chemicals extracted from diesel exhaust particles. J Immunol.

[b25-ehp-117-475] Kaewamatawong T, Shimada A, Okajima M, Inoue H, Morita T, Inoue K (2006). Acute and subacute pulmonary toxicity of low dose of ultrafine colloidal silica particles in mice after intratracheal instillation. Toxicol Pathol.

[b26-ehp-117-475] Khalil MAK, Rasmussen RA (2003). Tracers of wood smoke. Atmos Environ.

[b27-ehp-117-475] Kodavanti U, Schladweiler M, Ledbetter A, Hauser R, Samet J, McGee J (2002). Pulmonary and systemic effects of zinc-containing emission particles in three rat strains: multiple exposure scenarios. Toxicol Sci.

[b28-ehp-117-475] Laden F, Neas LM, Dockery DW, Schwartz J (2000). Association of fine particulate matter from different sources with daily mortality in six U.S. cities. Environ Health Perspect.

[b29-ehp-117-475] Li N, Sioutas C, Cho A, Schmitz D, Misra C, Sempf J (2003). Ultrafine particulate pollutants induce oxidative stress and mitochondrial damage. Environ Health Perspect.

[b30-ehp-117-475] Lin M, Chen Y, Burnett RT, Villeneuve PJ, Krewski D (2002). The influence of ambient coarse particulate matter on asthma hospitalization in children: case-crossover and time-series analyses. Environ Health Perspect.

[b31-ehp-117-475] Mar TF, Larson TV, Stier RA, Claiborn C, Koenig JQ (2004). An analysis of the association between respiratory symptoms in subjects with asthma and daily air pollution in Spokane, Washington. Inhal Toxicol.

[b32-ehp-117-475] Mar T, Norris G, Koenig J, Larson T (2000). Associations between air pollution and mortality in Phoenix, 1995–1997. Environ Health Perspect.

[b33-ehp-117-475] Marcazzan GM, Vaccaro S, Valli G, Vecchi R (2001). Characterisation of PM_10_ and PM_2.5_ particulate matter in the ambient air of Milan (Italy). Atmos Environ.

[b34-ehp-117-475] McConnell R, Berhane K, Gilliland F, Molitor J, Thomas D, Lurmann F (2003). Prospective study of air pollution and bronchitic symptoms in children with asthma. Am J Respir Crit Care Med.

[b35-ehp-117-475] Miller M, Marty M, Arcus A, Brown J, Morry D, Sandy M (2002). Differences between children and adults: implications for risk assessment at California EPA. Int J Toxicol.

[b36-ehp-117-475] Naeher LP, Brauer M, Lipsett M, Zelikoff JT, Simpson CD, Koenig JQ (2007). Woodsmoke health effects: a review. Inhal Toxicol.

[b37-ehp-117-475] Nel A, Diaz-Sanchez D, Li N (2001). The role of particulate pollutants in pulmonary inflammation and asthma: evidence for the involvement of organic chemicals and oxidative stress. Curr Opin Pulm Med.

[b38-ehp-117-475] Nel A, Diaz-Sanchez D, Ng D, Hiura T, Saxon A (1998). Enhancement of allergic inflammation by the interaction between diesel exhaust particles and the immune system. J Allergy Clin Immunol.

[b39-ehp-117-475] Ostro B, Broadwin R, Green S, Feng WY, Lipsett M (2006). Fine particulate air pollution and mortality in nine California counties: results from CALFINE. Environ Health Perspect.

[b40-ehp-117-475] Ostro B, Feng WY, Broadwin R, Green S, Lipsett M (2007). The effects of components of fine particulate air pollution on mortality in California: results from CALFINE. Environ Health Perspect.

[b41-ehp-117-475] Peel JL, Tolbert PE, Klein M, Metzger KB, Flanders WD, Todd K (2005). Ambient air pollution and respiratory emergency department visits. Epidemiology.

[b42-ehp-117-475] Pope CA (1989). Respiratory disease associated with community air pollution and a steel mill, Utah Valley. Am J Public Health.

[b43-ehp-117-475] Pritchard RJ, Ghio AJ, Lehman JR, Winsett DW, Tepper JS, Park P (1996). Oxidant generation and lung injury after particulate air pollutant exposure increases with the concentrations of associated metals. Inhal Toxicol.

[b44-ehp-117-475] R Development Core Team (2006). R: A Language and Environment for Statistical Computing.

[b45-ehp-117-475] Rubin JI, Brown SG, Wade KS, Hafner HR (2006). Apportionment of PM_25_ and air toxics in Detroit, Michigan. Final report prepared for the U.S. Environmental Protection Agency, Research Triangle Park, NC.

[b46-ehp-117-475] Salvi S, Blomberg A, Rudell B, Kelley F, Sandstrom T, Holgate S (1999). Acute inflammatory responses in the airways and peripheral blood after short-term exposure to diesel exhaust in healthy human volunteers. Am J Respir Crit Care Med.

[b47-ehp-117-475] Sarnat JA, Marmur A, Klein M, Kim E, Russell AG, Sarnat SE (2008). Fine particle sources and cardiorespiratory morbidity: an application of chemical mass balance and factor analytical source-apportionment methods. Environ Health Perspect.

[b48-ehp-117-475] Schauer J, Cass G (2000). Source apportionment of wintertime gas-phase and particle-phase air pollutants using organic compounds as tracers. Environ Sci Technol.

[b49-ehp-117-475] Schauer J, Lough G, Shafer M, Christensen W, Arndt M, DeMinter J (2006). Research Report: Characterization of Metals Emitted from Motor Vehicles.

[b50-ehp-117-475] Schlesinger RB, Kunzli N, Hidy GM, Gotschi T, Jerrett M (2006). The health relevance of ambient particulate matter characteristics: coherence of toxicological and epidemiological inferences. Inhal Toxicol.

[b51-ehp-117-475] Schwartz J (2004). Air pollution and children’s health. Pediatrics.

[b52-ehp-117-475] Schwartz J, Dockery DW, Neas LM (1996). Is daily mortality associated specifically with fine particles?. J Air Waste Manag Assoc.

[b53-ehp-117-475] Sinclair AH, Tolsma D (2004). Associations and lags between air pollution and acute respiratory visits in an ambulatory care setting: 25-month results from the aerosol research and inhalation epidemiological study. J Air Waste Manag Assoc.

[b54-ehp-117-475] Tolbert P, Mulholland J, MacIntosh D, Xu F, Daniels D, Devine O (2000). Air quality and pediatric emergency room visits for asthma in Atlanta, Georgia, USA. Am J Epidemiol.

[b55-ehp-117-475] van der Zee S, Hoek G, Boezen H, Schouten J, van Wijnen J, Brunekreef B (1999). Acute effects of urban air pollution on respiratory health of children with and without chronic respiratory symptoms. Occup Environ Med.

[b56-ehp-117-475] World Health Organization (1975). International Classification of Diseases.

[b57-ehp-117-475] Zanobetti A, Schwartz J, Gold DR (2000). Are there sensitive subgroups for the effects of airborne particles?. Environ Health Perspect.

[b58-ehp-117-475] Zeka A, Zanobetti A, Schwartz J (2005). Short term effects of particulate matter on cause specific mortality: effects of lags and modification by city characteristics. Occup Environ Med.

[b59-ehp-117-475] Zelikoff JT, Schermerhorn KR, Fang K, Cohen MD, Schlesinger RB (2002). A role for associated transition metals in the immunotoxicity of inhaled ambient particulate matter. Environ Health Perspect.

